# Health-related quality of life in adults with hematological cancer: a 2023 cross-sectional survey from Qatar

**DOI:** 10.3389/fonc.2024.1391429

**Published:** 2024-05-28

**Authors:** Yasamin Abdu, Khalid Ahmed, Mariam Abdou, Rayan Elhussein, Sayda Sirelkhatim, Iheb Bougmiza, Mohamed A. Yassin, Nagah A. Selim

**Affiliations:** ^1^ Community Medicine Department, Hamad Medical Corporation, Doha, Qatar; ^2^ Department of Hematology, Royal Hallamshire Hospital, Sheffield Teaching Hospitals NHS Foundation Trust, Sheffield, United Kingdom; ^3^ MBBS, Faculty of Medicine, Al Mugtaribeen University, Khartoum, Sudan; ^4^ MBBS, Faculty of Medicine, Alzaiem Alazhari University, Khartoum, Sudan; ^5^ Community Medicine Department, Primary Health Care Corporation, Doha, Qatar; ^6^ Department of Hematology, National Center for Cancer Care and Research (NCCCR), Hamad Medical Corporation, Doha, Qatar

**Keywords:** health-related quality of life, quality of life, depressive symptoms, hematological cancer, blood cancer

## Abstract

**Background:**

Hematological cancers impose a complex burden on individuals, affecting their physical health and mental and emotional well-being. This study evaluated the health-related Quality of Life (HRQOL) and its determinants among adults with hematological cancers in Qatar in 2023.

**Methods:**

A cross-sectional study used a validated structured questionnaire conducted among adult patients diagnosed with hematological cancers. All adult patients who attended The National Centre for Cancer Care and Research (NCCCR) in Qatar during the Data collection period (January to March 2023) and agreed to participate were included in the study.

**Results:**

A total of 257 participants were enrolled in the study. The highest median (IQR) score of the HRQOL domain was observed in the functionating score of 90.6 (13.8), followed by the global health score of 83.3(25. 0). The median (IQR) of the symptoms burden score was 07.4(12.3). Gender significantly affects HRQOL, with males reporting better functioning and lower symptom burden than females. Employment status is positively associated with functioning scores. Regular exercise correlates with higher global health and functioning scores and lower symptom burden, while depressive symptoms are linked to poorer HRQOL outcomes. Patients experiencing cancer recurrence or active disease report lower global health and functioning scores and higher symptom burden. Treatment modalities such as chemotherapy and bone marrow transplant (BMT) timing also influence HRQOL, with recent treatment recipients showing lower global health and higher symptom burden scores. Depressive symptoms were the primary factor, lowering the global health score by 15.2%. Regarding the low functioning score predictors, female gender, depressive symptoms, and cancer recurrence emerged as significant predictors of the low functioning score. Furthermore, Regular exercise increased the functioning score by 03.4 units (p-value=0.018). Finally, Multiple linear regression analysis reinforced the significance of depressive symptoms, active disease status, and recurrence within the past five years as substantial predictors of higher symptom scores.

**Conclusions:**

The study emphasizes the profound impact of depressive symptoms on all aspects of Health-Related Quality of Life (HRQOL), mainly affecting global health. It highlights the positive role of regular exercise in enhancing global health, functioning, and symptom burden scores.

## 1 Introduction

Cancer, including hemtological cancers, remains a leading cause of death globally, with projections indicating a further increase in cancer-related mortality in the years to come. Hematological cancers encompass a variety of diseases, including Hodgkin’s lymphoma, non-Hodgkin’s lymphoma, leukemia, and multiple myeloma. Leukemia, for instance, encompasses several subtypes, such as acute myeloid leukemia, chronic myelogenous leukemia, acute lymphoblastic leukemia, and chronic lymphoblastic leukemia. Significantly, these diseases can affect individuals of all ages, with a tendency for increased incidence as individuals grow older (1).

Hematological cancers, like non-Hodgkin lymphoma and leukemia, are globally significant, ranking among the most common and deadliest cancers in 2020. That year, non-Hodgkin lymphoma was the 11th most frequently diagnosed cancer, while leukemia was the 13th, yet both stood as the 10th and 11th leading causes of cancer-related deaths, respectively (2). Despite the potential for high five-year survival rates of up to 95%, concerns persist regarding the Quality of life for those affected (3). In Saudi Arabia, leukemia notably topped the list of hematological cancers in 2014, indicating its significant impact within the country (4). Similarly, in Qatar, data from both the World Health Organization (5) and the Ministry of Public Health (6) underscored the prevalence of leukemia and non-Hodgkin lymphoma. According to these sources, leukemia was the fourth most common cancer overall, while non-Hodgkin lymphoma ranked seventh across all genders and nationalities.

HRQOL refers to the physical, psychological, and social domains of health seen in areas influenced by a person’s experiences, beliefs, expectations, and perceptions (7). However, measuring HRQOL poses challenges due to its subjective nature, with interpretations varying widely among individuals. In the context of hematological cancer treatment, HRQOL becomes a significant concern to the severity of symptoms as well as the long duration of treatment, including a long inpatient treatment period for those who receive bone marrow transplantation, the medication sides effects like nausea, vomiting, anxiety, depression, psychological distress, dyspnea, fatigue, pain, neutropenia and insomnia which can impose a change in the patient function and the remitting and relapsing course of some hematological cancers. Moreover, Hematological cancers often necessitate aggressive treatments such as surgery, chemotherapy, and radiotherapy. Unfortunately, these treatments can bring about significant physical and bodily side effects. For instance, high-dose chemotherapy and/or total body irradiation can lead to hair loss, disfigurement, alterations in body weight, growth impairment, and restrictions in physical mobility. These adverse effects can contribute to feelings of discomfort regarding body image among survivors of hematological cancer (8). So, all these factors can affect not only quality of life and work status but also family life and the prognosis and treatment outcome (9).

An in-depth analysis of the literature reveals the impairment of HRQOL impairment in patients with hematological cancers. Physical symptoms, including fatigue (10), pain (11), and insomnia (12), exert profound effects on daily functioning, while psychological distress, such as anxiety and depression, exacerbates emotional burdens during treatment phases (13, 14). Moreover, cognitive impairments (15) and social functioning (14–16) deficits further underscore these malignancies’ comprehensive toll on patients’ overall well-being.

Therefore, providing blood cancer patients with comprehensive support, including counselling, social support, financial assistance, and high-quality medical care delivered by multidisciplinary teams is imperative (17). Studying the HRQOL among these patients can improve treatment outcomes and individual survival rates.

Up to our knowledge, there is limited data regarding HRQOL among blood cancer patients in the Gulf countries, particularly in Qatar. Thus, this study aims to bridge this gap by evaluating the HRQOL of hematological cancer patients, focusing on their physical, emotional, and social well-being. We hypothesized several correlations regarding hematological cancer patients’ overall health and well-being. Firstly, we anticipated that those who exercise regularly would demonstrate higher global health, functioning, and symptom burden domain scores than their sedentary counterparts. Secondly, patients with hematological cancer who are actively undergoing treatment are anticipated to display lower scores across various HRQOL domains compared to individuals who are in remission. Additionally, we hypothesized that patients in advanced disease stages would have lower scores than early-stage patients and that patients receiving chemotherapy for less than a year would have lower scores than those receiving chemotherapy for longer durations. By identifying the relationships between sociodemographic factors, health-related variables, disease characteristics, and HRQOL impairment, we can better understand the challenges faced by these patients. This research is crucial for assessing social and clinical risks and identifying intervention targets to enhance the QOL of hematological cancer patients in Qatar and beyond. Additionally, this research will serve as a foundation for future research at a national level, ultimately contributing to improved care and outcomes for individuals affected by blood cancers.

## 2 Methods

### 2.1 Study design and study setting

An analytical Cross-sectional study was utilized. This study was carried out in Hematology clinics and Daycare units at NCCCR, which is part of Hamad Medical Corporation (HMC) and looks after cancer patients and patients with hematological problems (18). All hematological cancer patients are treated and followed up in the NCCCR. The health services these clinics provide are either subsidised or free to the entire registered population, nationals, and expatriates.

### 2.2 study population and procedure

After obtaining approval from the Institutional Review Board (IRB) of Hamad Medical Corporation (MRC-01–22-530), the daily clinic attendance list was reviewed to identify eligible participants based on the study criteria. Eligible individuals aged 18 to 65, diagnosed with various types of hematological cancers and receiving treatment at The NCCCR during the study period (January to March 2023), were approached. Criteria for inclusion also encompassed the ability to read and write in either Arabic or English, along with a willingness to provide written informed consent. Exclusion criteria applied to vulnerable subjects such as pregnant individuals and those with cognitive or mental health issues (excluding depression) that could potentially affect communication or influence scores in different domains of HRQOL. Following identification, eligible participants underwent an orientation session, where the study’s details were explained, and written informed consent was obtained, emphasizing their right to refuse participation. Interviews and questionnaire administration occurred on the same day as their clinic appointments. They were conducted in a private room away from clinical areas by either the principal investigator or designated delegates.

### 2.3 Research instruments

For the sociodemographic and health-related data, an interview was conducted using a structured questionnaire after reviewing the relevant literature to collect the data. These data included age in years, gender of the participant, marital status, living condition, educational status, employment status, regular exercise, smoking status, alcohol status. and the presence of other comorbidities. Depressive symptoms were assessed by a validated questionnaire, the Patient Health Questionnaire (PHQ-9), and a cut-off point ≥4 was used to indicate significant depressive symptoms. it has an excellent internal validity, with Cronbach a of 0.089 (19).

A data extraction sheet was used to collect data such as body mass index (BMI)and hematological cancer-related data from the patient’s medical records, which were confirmed by attending hematology consultants and fellows.

After the permission of the developing organization, the HRQOL was assessed by a self-administered and pre-validated questionnaire, the European Organization for Research and Treatment of Cancer Quality of Life Questionnaire (EORTC QLQ-C30) version 3. EORTC QLQ-C30 is a disease-specific questionnaire to evaluate the quality of life in cancer patients. The 30 questions in the questionnaire comprised different scales, which included: the five functional scales, which measure physical (5 items), role(2 items), cognitive(2 items), emotional (4 items), and social functioning(2 items); the five symptoms scale that measure fatigue(3 items), pain(2 items), and nausea or vomiting(2 items); 1 global health status scale(2 items); and a six single-item scale that measures dyspnea, insomnia, appetite loss, constipation, diarrhea, and financial difficulties.

The questions were rated on a scale of 4 points: “Not at all”, “A little”, “Quite a bit”, or “Very much”. On the other hand, global health status was rated on a 7-point scale that ranged from “Very poor” to “Excellent”. Each scale was given its total score, which ranged from 0–100 and was computed from the raw scores and was transformed linearly by referring to the EORTC QLQ-C30 scoring manual (20).

Besides the global health QOL scale, we followed the suggestions of Hinz et al. (21) to form two further comprehensive scales by averaging the scores across all functioning scales (functioning) and symptom scales (symptoms burden). These scales ‘ internal consistencies (Cronbach´s Alpha) were good (α global = .89; α functioning = .93; α symptoms = .90). For the global health and functioning domain scores, higher scores represented better global health status and better functioning; however, for the symptom burden score, higher scores were indicative of more symptoms (21). Overall, the questionnaire took approximately 10–15 minutes to complete.

### 2.4 Outcome variable

HRQOL was assessed as a continuous variable using the EORTC QLQ-C30. Each scale will be given its total score, which ranges from 0–100 and will be computed from the raw scores and will be transformed linearly by referring to the EORTC QLQ-C30 scoring manual; besides the global health QOL scale, we formed two further comprehensive scales by averaging the scores across all functioning scales (functioning) and symptom scales (symptoms burden). For the global health and working domain scores, higher scores represented better global health status and better functioning; however, for the symptom burden score, higher scores were indicative of more symptoms (20).

### 2.5 Statistical analysis

Statistical Package for the Social Sciences (SPSS) version 26 was used to manage and analysis the data. Continuous data were tested for normality, and descriptive statistics were conducted. As our dependent variable (HRQOL) was not normally distributed, the Mann-Whitney test assessed the associations between the independent variables and the total scores for global health, functioning, and symptom burden. A p-value equal to or less than 0.05 was considered statistically significant and further included in the multivariate analysis. Finally, multiple linear regression was used to assess the predictors of global health, functioning, and symptom burden domain scores.

## 3 Results

### 3.1 Description of the study population

In this cross-sectional study, we approached all adult patients (18–65 years) diagnosed with hematological cancers at the NCCCR during our data collection period (280). Out of these, 257 patients agreed to participate, with a response rate of 91.8%.

**Figure 1:**
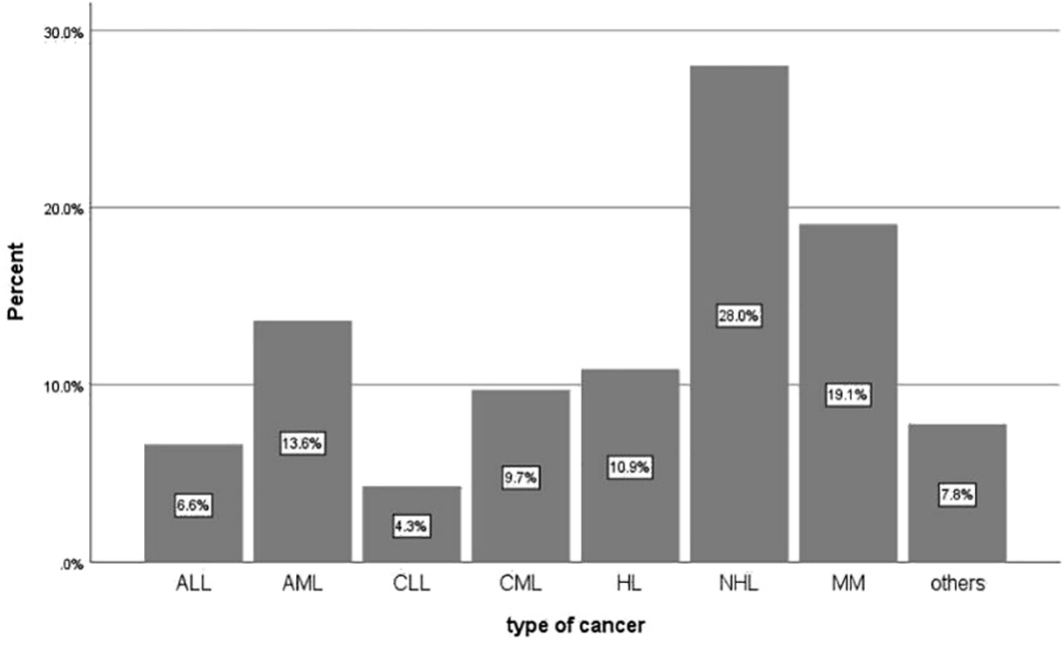
Types of Hematological Cancer among Adult Patients with Hematological cancer at the NCCCR in Qatar during 2023 (*N* = 257).

The most common cancer type was non-Hodgkin lymphoma 72(28.0%), followed by multiple myeloma, Acute myeloid leukemia, and Hodgkin lymphoma 49.0(19.1%), 35.0(13,6%), and 28.0(10.9%), respectively, as shown in **Figure 1**.

The mean (± SD) age of the study’s participants was 45.59 (± 11.6). Males constituted the majority, accounting for 63.8% of the sample. Additionally, 81.7% were married, and 61.1% lived with their families. Regarding education, over half (54.9%) held university and postgraduate degrees, while the majority (72.7%) were employed.

Most participants (62.6%) were overweight or obese. Additionally, 66.1% of hematological cancer patients admitted to leading a sedentary lifestyle without regular exercise, while 33.1% reported a history of smoking. Moreover, a small minority (6.6%) acknowledged past alcohol consumption. In terms of current health issues, 98 (38.1%) reported concurrent chronic illnesses, with hypertension being the most prevalent (58, 22.6%), followed by Diabetes Mellitus (41, 16%).

Furthermore, a significant portion of patients (72.4%) received their cancer diagnosis within the last five years, with 42.3% presenting at an advanced stage (stage IV). Among these, a majority (72.4%) had an active disease, while 16.7% experienced recurrence within the past five years. Regarding treatment history, 55.6% were undergoing chemotherapy at the time of the study. Most patients (69.2%) initiated their chemotherapy within the past year. Moreover, 23.0% of patients underwent Bone Marrow Transplantation, with 42.4% of these occurring within the last year. Additionally, a small subset (6 participants, 2.3%) had coexisting cancers, such as osteosarcoma, as seen in **Table 1**.

**Table 1:** The sociodemographic, Health-related, and Hematological–related Characteristics of adult patients with hemtological Cancer at the NCCCR in Qatar2023(n=257).

Variable	Frequency (Percentage %)
Sociodemographic characteristics	
Age: Mean age ± (SD) Male Married Living with a family member University and Higher education level Employed status*^(n=249)^	45.59 ± 11.63 164(63.8%) 210(81.7%) 157(61.1%) 141(54.9%) 181(72.7%)
Health-related characteristics	
Overweight/obese Regular exercise Ever smoking history Ever alcohol history Comorbidities	161(62.6%) 87(33.9%) 85(33.1%) 17(06.6%) 98(38.1%)
Hematological cancer-related characteristics	
Diagnosis time (≤5 years) Stage IV ^* * (n=163)^ Recurrence in the past five years Currently in active disease Current chemotherapy One year or less since starting chemotherapy **^(n=143)^** Received BMT One year or less since BMT**^(n=59)^**	186(72.4%) 69(42.3%) 43(16.7%) 186(72.4%) 143(55.6%) 30(20.7%) 95(23.0%) 25(42.4%)

^*^ 8 participants (3.1%) refused to report their employment status, ^**^ 94(36.3%) participants had no stage recorded as their hematological cancer type has no international staging; BMT, Bone Marrow Transplantation.

### 3.2 HRQOL domain scores

**Figure 2:**
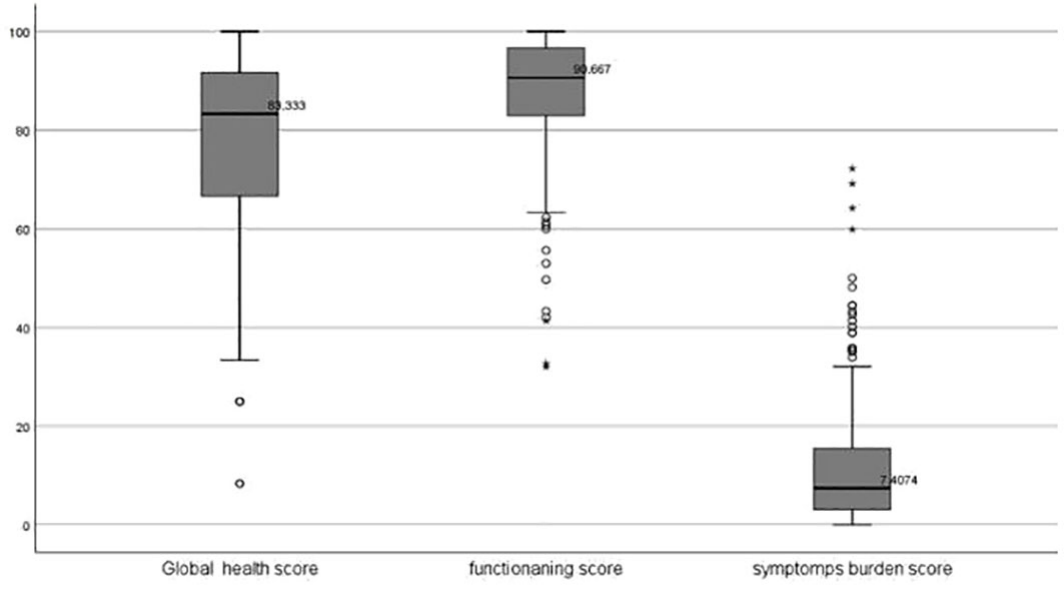
The Box plots for the Distribution of the global health, functionating and symptoms burden scores of the HRQOL among adult patients with hematological cancers in Qatar 2023.

Regarding the HRQOL domain scores, the highest median (IQR) score was observed in the functionating score, 90.6 (13.8), followed by the global health score, 83.3(25. 0). The median (IQR) of the symptoms burden score was 7.4(12.3), where the lower score indicates fewer symptoms and better quality of life, as seen in **Figure 2**.

### 3.3 Associations between the independent variable and HRQOL domain scores

Statistical analysis revealed significant associations (p-value <0.05) between certain socio-demographic factors and HRQOL domain scores. Specifically, gender was found to significantly influence both Functioning Score and symptom burden Score, with males reporting higher functioning and lower symptom burden than females with a median (IQR) of [92.0(12.0) vs 87.0(17.17)] and [6.8(11.6) vs 9.87(17.59)], respectively. However, no significant differences were observed based on marital status or living arrangements. Moreover, employment status was associated with the functioning score, with employed individuals exhibiting a better functioning score than their non-employed counterparts, with a p-value of 0.012, as shown in **Table 2**.

**Table 2:** The Associations between Sociodemographic characteristics and the total score of the HRQOL domain among adult patients with hematological Cancer at the NCCCR in Qatar 2023.

	Global Health score		Functioning Score		Symptoms burden score	
Sociodemographic characteristic	Median (IQR)	P value	Median (IQR)	P value	Median (IQR)	P value
Gender						
Males Females	83.3 (25.0) 83.3 (33.3)	0.563	92.0 (12.0) 87.0 (17.2)	**0.001**	06.8 (11.6) 09.9 (17.6)	**0.008**
Marital status						
Single Married	83.3 (25.0) 83.3 (25.0)	0.714	89.3 (12.7) 90.7 (14.0)	0.415	07.4 (16.7) 07.7 (11.4)	0.914
Living with a family member						
Yes No	83.3 (33.3) 75.0 (25.0)	0.384	90.6 (13.3) 90.2 (14.2)	0.769	08.0 (12.3) 07.4 (14.9)	0.615
Employment status ^(n=249)^						
Employed Non-employed	83.3 (25.0) 83.3 (25.0)	0.873	91.7 (13.2) 87.8 (19.3)	**0.012**	07.4 (11.4) 09.6 (18.4)	0.082

Bold values indicate statistically significant p value< 0.05.

Statistically significant associations (p-value < 0.05) were found between several health-related characteristics and HRQOL domain scores. Notably, patients who reported engaging in regular exercise demonstrated significantly higher scores in the global health and functioning domain and lower symptom burden scores than those who did not exercise (p = 0.001, p < 0.001, p = 0.005, respectively). Conversely, patients with a history of depressive symptoms exhibited notably lower scores in the global health and functioning domain and higher symptom burden scores than those without depressive symptoms (p < 0.001 for all domains). No significant associations were found between HRQOL domain scores and overweight/obesity, smoking history, or comorbidities, as shown in **Table 3**.

**Table 3:** The Associations between Health-related characteristics and the total score of the different domains of HRQOL among adult patients with hematological Cancer at the NCCCR in Qatar 2023.

	Global Health score		Functioning Score		Symptoms burden score	
Health-related characteristic	Median (IQR)	P value	Median (IQR)	P value	Median (IQR)	P value
Overweight/obesity						
Yes No	83.3 (33.3) 75.0 (16.7)	0.086	90.6 (12.6) 89.7 (17.0)	0.477	07.4 (10.5) 09.2 (16.4)	0.363
Regular exercise						
Yes NO	83.3 (25.0) 75.0 (25.0)	**0.001**	93.3 (14.7) 89.3 (15.8)	**<0.001**	05.5 (11.7) 08.0 (14.8)	**0.005**
Ever smoking history						
Yes No	83.3 (25.0) 83.3 (25.0)	0.661	90.7 (12.2) 90.5 (15.2)	0.931	07.4 (10.5) 07.7 (13.3)	0.857
Comorbidities						
Yes No	83.3 (33.3) 75.0 (25.0)	0.146	91.8 (11.4) 90.0 (16.3)	0.625	07.4 (12.8) 08.0 (11.7)	0.766
Depressive symptoms						
Yes No	58.3 (29.17) 83.3 (31.2)	**<0.001**	75.7 (19.0) 93.3 (11.6)	**<0.001**	24.1 (25.3) 05.5 (09.9)	**<0.001**

Bold values indicate statistically significant p value< 0.05.

**Table 4:** The Associations between Hematological cancer-related characteristics and the total score of the different domains of HRQOL among adult patients with hematological Cancer at the NCCCR in Qatar 2023.

	Global Health score		Functioning Score		Symptoms burden score	
Hematological cancer-related characteristic	Median (IQR)	P value	Median (IQR)	P value	Median (IQR)	P value
Time Since the diagnosis						
Less than five years Five years or more	83.3 (25.0) 83.3 (33.3)	0.239	90.7 (13.4) 90.0 (18.0)	0.979	08.0 (13.6) 07.4 (12.3)	0.391
Stage of haematological cancer ^(n=163)^						
Stage IV Other less severe stages	75.0 (29.2) 83.3 (25.0)	0.793	89.3 (13.5) 92.0 (11.6)	0.203	8.02 (13.9) 8.02 (09.3)	0.621
Recurrence in the past five years						
Yes No	66.7 (25.0) 83.3 (33.3)	**0.009**	84.0 (18.7) 91.7 (13.1)	**<0.001**	12.3 (15.4) 07.4 (11.7)	**0.001**
Currently in active disease						
Yes No	83.3 (25.0) 83.3 (33.3)	0.185	90.0 (14.6) 91.7 (13.0)	0.064	08.6 (14.8) 06.8 (11.1)	**0.007**
Current chemotherapy						
Yes No	83.3 (25.0) 38.3 (33.3)	0.233	90.0 (15.3) 91.0 (12.1)	0.331	8.6 (14.8) 6.7 (10.6)	**0.030**
Time since the current chemotherapy						
**≤1 year** **>1 year**	75.00 (25.0) 83.3 (27.1)	**0.005**	93.3 (16.7) 89.0 (14.1)	0.052	9.8 (15.6) 4.9 (11.1)	**0.012**
Received BMT						
**Yes** **No**	75.0 (27.1) 83.3 (25.0)	0.222	87.7 (15.5) 90.7 (13.0)	0.100	8.0 (11.4) 7.4 (12.3)	0.976
Time since BMT						
**≤1 year** **>1 year**	66.7 (18.7) 83.3 (37.5)	**0.038**	87.3 (37.7) 92.0 (20.1)	0.435	8.9 (11.6) 7.4 (12.9)	0.501

Bold values indicate statistically significant p value< 0.05.

With respect to the association between the hematological cancer-related characteristics and HRQOL domain scores, patients experiencing recurrence within the past five years demonstrated significantly lower scores in the global health and functioning domain and higher symptom burden scores compared to those without recurrence (p = 0.009 for Global Health score, p < 0.001 for Functioning Score, and p = 0.001 for Symptoms Burden score). Moreover, a statistically significant association(p-value=0.007) was found between being in active disease and the symptom burden domain score, with a higher score for those currently in active disease. The median (IQR) symptom burden domain scores were 08.6 (14.8) and 06.8(11.1) for the patients with and without active disease, respectively. Conversely, no significant associations were observed between HRQOL domain scores and the stage of hematological cancer.

Regarding the associations between the treatment received and HRQOL domain scores, patients who were on chemotherapy treatment had a higher symptom burden score compared to their counterparts who were not on chemotherapy, with a median (IQR)symptom burden domain score of 8.6(14.8) and 06.8(10.6), respectively, with a p-value of 0.030.

Patients who started chemotherapy within the last year had significantly lower global health and higher symptom burden scores than their counterparts who began chemotherapy more than one year ago, with a p-value of 0.005 and 0.012, respectively. Similarly, receiving BMT within the last year was significantly associated with a lower global health score,66.7(66.7), than receiving BMT more than one year ago,83.3(35.4), p-value =0.038, as seen in **Table 4**.

### 3.4 Predictors of the HRQOL domain scores

**Table 5** summarizes the simple and multiple linear regression results to assess the global health score predictors. After Adjusting for the significant variables from the bi-variate analysis, only the presence of depressive symptoms remained a statistically significant predictor of the Global Health Score; in fact, the presence of depressive symptoms decreased the global health score by 15.2%, with a p-value of 0.049. On the other hand, other variables, such as exercise, recurrence, time since starting chemotherapy, and time since receiving a BMT, were not statistically significant predictors when considered together while accounting for confounding factors. Moreover, the overall model is significant, with a p-value of 0.017.

**Table 5:** Multiple Linear Regression to assess the Predictors of The Global Health Score among adult patients with hematological cancer at the NCCCR in Qatar2023(N=257).

	Global health score					
	Unadjusted analysis			Adjusted analysis		
Explanatory Variable	β	Coefficient (95% CI)	P- value	β ^†^	Coefficient (95% CI)	P- value
Exercise (Yes)	08.4	(03.5 to 13.4)	0.001	-01.1	(-16.2 to 13.9)	0.881
Depressive symptoms (Yes)	-21.9	(- 26.9 to -16.9)	**<0.001**	-15.2	(-30.4 to -00.1)	**0.049**
Recurrence during the last 5 years (Yes)	-08.5	(-14.9 to -02.2)	0.009	-03.7	(-21.6 to 14.1)	0.669
Time since starting the current treatment (Within one year or less)	-07.6	(-14.0 to -01.2)	0.020	-20.6	(-41.9 to 00.8)	0.058
Time since receiving the BMT (One year or less)	-09.7	(-19.3 to -00.2)	0.046	04.2	(-15.1 to 23.6)	0.654
Constant				95.69		**<0.001**

CI, confidence interval; β, Non-adjusted beta coefficient; β^†^, Adjusted beta coefficient; BMT, Bone Marrow Transplantation. Bold values indicate statistically significant p value< 0.05.

Similarly, we found that being female was associated with a decrease of 3.2 units in the functioning score, on average, compared to males, and this result is statistically significant (p =0.021). Likewise, having depressive symptoms was significantly associated with a substantial decrease of 16.7 units in the functioning score, p-value < 0.001. Experiencing a recurrence of cancer in the last five years was associated with a reduction of 7.0 units in the functioning score, p-value <0.001. On the other hand, engaging in regular exercise was significantly associated with an increase of 3.4 units in the functioning score, p-value=0.018. The overall regression model was significant, with a p-value of < 0.001, as seen in **Table 6**.

**Table 6:** Multiple Linear Regression to assess the Predictors of the Functioning Score among adult patients with hematological cancer at the NCCCR in Qatar2023.

	Functioning score					
	Unadjusted analysis			Adjusted analysis		
Explanatory Variable	β	Coefficient (95% CI)	P- value	β ^†^	Coefficient (95% CI)	P- value
Gender (Female)	-05.2	(-08.4 to -02.0)	**0.001**	-03.3	(-06.1 to -00.4)	**0.021**
Employment (Yes)	04.5	(01.1 to 07.9)	**0.010**	-00.2	(-03.4 to 02.8)	0.987
Regular exercise (Yes)	05.5	(02.3 to 08.7)	**0.001**	03.4	(00.7 to 06.1)	**0.018**
Depressive symptoms (Yes)	-17.9	(-20.8 to -14.9)	**<0.001**	-16.7	(-15.69 to -10.42)	**<0.001**
Recurrence during the last 5 years (Yes)	-06.8	(-10.9 to -02.7)	**0.001**	-07.0	(-10.2 to -03.9)	**<0.001**
Constant				93.5		**<0.001**

CI, confidence interval; β, Non-adjusted beta coefficient; β^†^, Adjusted beta coefficient. Bold values indicate statistically significant p value< 0.05.

Finally, depressive Symptoms, recurrence during the last five years, and current active disease remain statistically significant predictors of the symptoms burden score. Having depressive symptoms had the most potent effect, increasing the score by 19.5 units, followed by being in currently active disease (an increase of 10.3 units) and recurrence during the Last five years (a rise of 3.9 units). However, gender, regular exercise, receiving current chemotherapy treatment, and time Since starting the current chemotherapy treatment were no longer statistically significant predictors of the symptoms burden score as their p-values were greater than 0.05, as seen in **Table 7**. The overall model is statistically significant with a p-value of <0.001.

**Table 7:** Multiple Linear Regression to assess the Predictors of the Symptoms Score among Adult Patients with Hematological Cancer at the NCCCR in Qatar2023.

	Symptoms burden score					
	Unadjusted analysis			Adjusted analysis		
Explanatory Variable	β	Coefficient (95% CI)	P- value	β ^†^	Coefficient (95% CI)	P- value
Gender (Female)	05.0	(01.8 to 08.3)	**0.002**	01.2	(-02.1 to 04.50)	0.488
Regular exercise (Yes)	-04.6	(-07.9 to -01.30	**0.006**	-00.5	(-03.8 to 02.8)	0.497
Depressive symptoms (Yes)	14.2	(12.3 to 16.0)	**<0.001**	19.5	(15.8 to 23.2)	**<0.001**
Recurrence during the last 5 years (Yes)	04.5	(00.2 to 08.7)	**0.038**	03.9	(00.5 to 08.0)	**0.050**
Current active disease (Yes)	04.7	(01.1 to 08.2)	**0.009**	10.3	(02.4 to 18.2)	**0.011**
Current chemotherapy(Yes)	02.7	(00.4 to 05.9)	**0.030**	01.4	(-02.6 to 05.5)	0.480
Time since receiving the current chemotherapy (≤1 year)	04.0	(00.2 to 08.4)	**0.020**	01.9	(-01.4 to 05.4)	0.262
Constant				-05.8		0.202

CI, confidence interval; β, Non-adjusted beta coefficient; β^†^, Adjusted beta coefficient. Bold values indicate statistically significant p value< 0.05.

## 4 Discussion

This study explored the factors influencing HRQOL among adult patients with hematological cancers. Significant associations were found between sociodemographic factors such as gender and employment status and HRQOL domain scores. Health-related characteristics like regular exercise and depressive symptoms also played a crucial role in determining HRQOL outcomes. Furthermore, hematological cancer-related factors, including cancer recurrence and active disease status, significantly impacted HRQOL. Moreover, regression analysis identified depressive symptoms as a consistent predictor of lower global health and functioning scores and higher symptom burden scores. A possible explanation is that Depressive symptoms can distort one’s perception of their health. Individuals with depression may be more inclined to focus on negative aspects of their health and well-being. At the same time, recurrence and active disease were associated with higher symptom burden scores, and Regular exercise increased the functioning score by 3.4 units. These findings highlight the importance of addressing psychological distress and disease management to enhance HRQOL among hematological cancer patients.

Similarly, a study conducted among 5339 cancer survivors, out of which 1751 patients had hematological cancer, found that female patients reported significantly lower functioning and higher symptom burden scores (22). Hence, it is crucial to address the differences in the experiences of male and female patients regarding the long-term physical symptoms of cancer and the impact on their functioning status. Despite the clear differences highlighted in our results, it is apparent that these gender-specific variations are not consistently considered when providing information, care, and counselling to cancer patients. It is essential to discuss these differences when making therapeutic decisions openly. While awareness of future symptoms may not prevent their occurrence, acknowledging them can empower patients and enhance their sense of control. Moreover, if requested, providing more detailed information could strengthen patients’ ability to manage the difficulties they encounter throughout their cancer journey (23, 24).

Consistent with our study, a randomized controlled trial from Canada (25) highlights the positive impact of exercise on global health outcomes, and it enhances cardiovascular fitness and improves physical functioning in patients with lymphoma. This similarity reinforces the evidence supporting physical activity as a crucial component of survivorship care, mitigating some treatment-related adverse effects and enhancing overall well-being. Reports suggest that physical activity could help slow down the spread of metastases (26), bolster the immune system through the support of natural killer cells (27), reduce low-grade inflammation and insulin resistance by stimulating the release of myokines through muscle contractions (28), boost cardiorespiratory health (29), and amplify the benefits of systemic treatments by enhancing the blood flow in the tumor microenvironment, thus reducing the severity of cancer characteristics (30). Moreover, exercise can prevent or reverse functional decline, manage symptoms, and uphold independence for individuals with advanced-stage diseases (31, 32).

This similarity reinforces the evidence supporting physical activity as a crucial component of survivorship care, mitigating some treatment-related adverse effects and enhancing overall well-being. Moreover, many professional cancer organizations recommend physical activity as a non-pharmacological treatment intervention for fatigue and pain in cancer populations (33, 34).

In line with our findings, a study conducted among 522 elderly patients (35), confirmed the negative impact of depressive symptoms on HRQOL domains. Depression causes a diminished QOL in cancer patients by worsening physical symptoms and increases the negative effect on patients and their families throughout the disease (36). Hence, health policies should facilitate the integration of mental health services within the cancer care system; this will help address depressive symptoms and promote emotional well-being as an integral part of cancer care.

Similarly, our findings align with multiple studies reporting pain (12, 37–40) and fatigue and a sense of reduced energy (41–43) as prevalent symptoms among hematological cancer survivors. Notably, a study (42) revealed that fatigue levels could remain elevated for years after treatment, which may be explained within the “sickness behavior” theoretical framework. This framework posits that the body will exhibit sickness behavior, such as fatigue, as a reaction to the body’s inflammatory response to fighting infection or a disease like cancer (44, 45). So, it is essential to address symptoms of pain and fatigue by developing strategies and interventions to manage these issues effectively, and it may involve medication, physical therapy, or lifestyle modifications.

Similar deterioration in HRQOL following chemotherapy treatment was reported in the study of patients with non-Hodgkin’s lymphoma (37). However, after chemotherapy, general health improved in non-Hodgkin’s lymphoma patients in one study (38). This discrepancy may be attributed to the type of chemotherapy and the timing of HRQOL assessments, as our study revealed that HRQOL was lower within one year of starting chemotherapy in the global health and the symptoms burden domain. Moreover, chemotherapy, while challenging, can lead to disease remission, which may positively influence patients’ perceptions of their global health over time.

In our study, patients who received BMT had lower global health and functioning scores and higher symptom burden scores; however, it was not statistically significant. However, the negative impact on the functional domain following BMT was noted in other studies (14, 46). One possible explanation is that the transplantation resulted in an immediate deterioration in HRQOL and an increase in symptom burden. These effects were reversed in the months following the procedure (47, 48). This parallels our findings, which indicate a statistically significant deterioration in global health within one year of BMT. Studies have shown that some symptoms, such as physical well-being (14), sleeping problems, pain (49), and anxiety were among the common factors affecting HRQOL after the BMT.

While we found active disease status to be associated with higher symptom burden, similarly, other studies have specifically linked it to compromised physical and mental health functioning (50), these findings suggest that the impact of active disease extends beyond physical symptoms to affect psychological domains of HRQOL, highlighting the multifaceted challenges patients face with ongoing disease activity. In line with our finding, a study involving 2001 survivors of hematological malignancies (51), who were assessed at least 2.5 years after their diagnosis and were sourced from two German cancer registries yielded similar results to our study, where they found no significant associations between Global Health, functioning, symptom burden scores, and factors such as cancer type and time since diagnosis. However, one study (12) identified an association between older age and a higher prevalence of pain, and another study (38) reported that patients with aggressive non-Hodgkin’s lymphoma experienced less pain during chemotherapy treatment. The later study (38) established an association between the severity of the cancer and the level of fatigue experienced by patients.

The clinical implications of the research underscore the critical need for policy interventions at the tertiary care level to address the multifaceted needs of individuals with hematological cancers. Tailored support programs must be developed, considering gender-specific needs, recurrence and active disease state, to ensure vulnerable subgroups receive essential assistance. Integration of mental health services within cancer care systems is imperative to address depressive symptoms and promote emotional well-being as integral components of care. Public health policies should promote physical activity among hematological cancer patients to enhance overall well-being, while strategies for pain management and reducing fatigue are crucial to alleviating distressing symptoms. Additionally, supporting patient advocacy groups can facilitate the advocacy for patients’ rights and needs, influencing policies and services to serve them better. Moreover, this research will serve as a foundation for future research at a national level, ultimately contributing to improved care and outcomes for individuals affected by blood cancers. Further research could explore the long-term effects of targeted interventions for depressive symptoms and the sustained benefits of incorporating exercise into supportive care plans. Additionally, investigating the impact of psychosocial support programs and alternative therapies on HRQOL could provide valuable insights into comprehensive strategies for managing hematological cancers. Such studies would help refine existing interventions and develop novel approaches to enhance the well-being of individuals facing these challenges.

One of the strengths of this study was the comprehensive data collection. The study utilized structured questionnaires and medical record data to collect various sociodemographic, health-related, and hematological cancer-related information. This comprehensive approach enabled a thorough analysis of the factors associated with the HRQOL domains. Moreover, The study used well-established instruments, the European Organization for Research and Treatment of Cancer Quality of Life Questionnaire (EORTC QLQ-C30), to assess HRQOL, which ensured the validity and reliability of the study’s measurements. Furthermore, the study was conducted at the National Centre for Cancer Care and Research (NCCCR), Qatar’s primary treatment for hematological cancer patients, to ensure the study’s generalizability. Nevertheless, the study had limitations, including a cross-sectional design that limited the ability to establish causality between variables. Moreover, The study relied on self-reported data for variables such as regular exercise, smoking history, and alcohol history. Self-reported data can be subjected to recall bias. Finally, the study required participants to read and write in Arabic or English. This language requirement might exclude individuals who are not proficient in these languages.

## 5 Conclusion

The overall quality of life reported was generally good, with high functioning and global health scores observed. However, this study underscores significant response variability in the symptoms burden domain, particularly regarding pain and fatigue, indicating areas requiring attention and specific interventions. Notably, depressive symptoms were found to strongly affect all HRQOL domains, especially global health perceptions. On a positive note, regular exercise emerged as a potential enhancer of global health and functioning, also contributing to alleviating symptom burden scores. These findings stress the importance of comprehensive strategies to address depressive symptoms through targeted interventions, while also highlighting the potential of exercise interventions to optimize HRQOL among individuals facing hematological cancers.

## Data availability statement

The raw data supporting the conclusions of this article will be made available by the authors, without undue reservation.

## Ethics statement

The studies involving humans were approved by Medical research Center(MRC-01-22-530). The studies were conducted in accordance with the local legislation and institutional requirements. The participants provided their written informed consent to participate in this study.

## Author contributions

YA: Conceptualization, Data curation, Formal analysis, Investigation, Methodology, Project administration, Resources, Software, Validation, Visualization, Writing – original draft, Writing – review & editing. KA: Conceptualization, Data curation, Investigation, Methodology, Writing – original draft, Writing – review & editing. MA: Data curation, Investigation, Software, Writing – original draft, Writing – review & editing. RE: Data curation, Investigation, Project administration, Software, Writing – original draft, Writing – review & editing. SS: Data curation, Software, Writing – original draft, Writing – review & editing. IB: Supervision, Writing – original draft, Writing – review & editing. MY: Conceptualization, Methodology, Project administration, Resources, Supervision, Writing – original draft, Writing – review & editing. NS: Conceptualization, Data curation, Methodology, Supervision, Writing – original draft, Writing – review & editing.
